# Un syndrome polyuro-polydipsique révélant une histiocytose langerhansienne

**DOI:** 10.11604/pamj.2015.22.27.7509

**Published:** 2015-09-11

**Authors:** Rachid Ammor, Assou Ajja

**Affiliations:** 1 Neurosurgery, Military Hospital My Ismail, Meknes, Morocco

**Keywords:** Syndrome polyuro-polydipsique, histiocytose langerhansienne, pathologies granulomateuses, Polyuro-polydipsique Syndrome, Langerhans cell histiocytosis, granulomatous diseases

## Image en medicine

Il s'agit d'un patient de 42 ans, admis pour des céphalées fronto-occipitales évoluant depuis six semaines et devenant de plus en plus rebelles au antalgiques usuels. Le patient a comme antécédents un diabète insipide central il y a 3 ans avec insuffisance antéhypophysaire sous traitement substitutif. L'enquête étiologique initiale était négative. L'examen clinique était sans particularité. Une radiographie du crane réalisée aux urgences a révélé des lacunes osseuses diffuses. La TDM cérébrale n'a pas objectivée de lésions intraparenchymateuses. Le patient a bénéficié d'une biopsie osseuse chirurgicale qui a confirmé le diagnostic de l'hystiocytose langerhansienne. (Prolifération d'allure histiocytaire sur un fond inflammatoire avec un immunomarquage positif PS100 et CD1a). Un bilan d'extension de la maladie a révélé une atteinte fémorale gauche ainsi qu'une atteinte splénique. Les histiocytoses langerhansiennes sont des pathologies granulomateuses rares, surtout de l'enfant, d’étiologie inconnue, d’évolution et de pronostic très variable ayant en commun une lésion histopathologique comportant une prolifération et une accumulation clonale de cellules ressemblant à la cellule de langerhans d'origine myélo-monocytaire. Le tableau clinique est hétérogène et dépend de la localisation et de l'intensité de l'infiltration. Le diabète insipide central est présent chez un peu prés 40% des patients avec atteinte systémique et peut même être révélateur de la maladie. Le traitement est nécessaire dans les formes multifocales pour réduire la morbimortalité.

**Figure 1:**
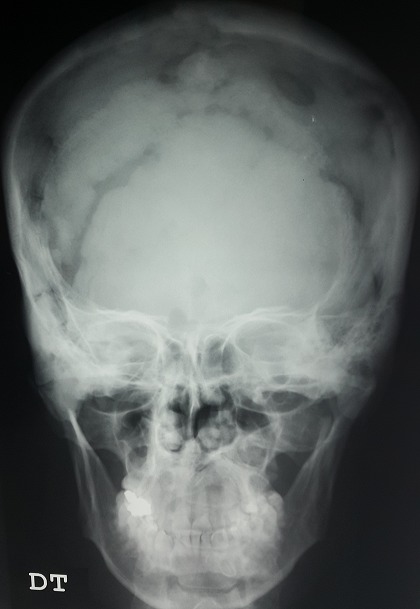
Radiographie standard crane de face montrant de multiples lésions lacunaires osseuses

